# Participation in the German mammography screening program: about 70% of women participate at least once between age 50 and 60

**DOI:** 10.1007/s10552-026-02278-2

**Published:** 2026-11-12

**Authors:** Miriam Heinig, Laura Buschmann, Florian Oesterling, Bianca Kollhorst, Jonas Czwikla, Heike Minnerup, André Karch, Ulrike Haug

**Affiliations:** 1 Department of Clinical Epidemiology, Leibniz Institute for Prevention Research and Epidemiology–BIPS, Achterstraße 30, 28359 Bremen, Germany; 2 Institute of Epidemiology and Social Medicine, University of Münster, Münster, Germany; 3 Cancer Registry North Rhine-Westphalia, Bochum, Germany; 4 Department of Statistical Methods in Epidemiology, Leibniz Institute for Prevention Research and Epidemiology–BIPS, Bremen, Germany; 5 Institute for Clinical Epidemiology and Biometry, Julius-Maximilians-Universität Würzburg, Würzburg, Germany; 6 Faculty of Human and Health Sciences, University of Bremen, Bremen, Germany

**Keywords:** Breast cancer, Mammography screening, Participation

## Abstract

**Purpose:**

The proportion of women participating in the German mammography screening program (MSP) is only 50% from a cross-sectional perspective. We aimed to determine longitudinal participation over 11 years (from age 50 to 60).

**Methods:**

We used cohort data with screening information from the cancer registry of North-Rhine Westphalia (NRW), covering the entire population of this federal state, and health claims data covering ~ 20% of the German population. We followed women born in 1959 from 2009 until 2019 and determined the proportion with ≥ 1 MSP participation. We repeated the analyses for other birth cohorts (1960–1962) and described participation patterns including age at first participation.

**Results:**

The proportion with ≥ 1 MSP participation was ~ 70%. This was consistent across both data sources for the 1959 birth cohort (NRW: 75.4%; health claims data: 71.0%) and across birth cohorts (1960: 70.4%, 1961: 70.1%, 1962: 69.7%). Among participating women, ~ 90% were between age 50 and 53 at first participation; 52.4% did not participate regularly.

**Conclusion:**

The proportion of women participating at least once in the MSP in 11 years is substantially higher compared to the proportion of invited women participating per year (cross-sectional perspective). Studies are required that explore reasons for irregular participation.

**Supplementary Information:**

The online version contains supplementary material available at https://doi.org/10.1007/s10552-026-02278-2.

## Introduction

Women between 50 and 75 years in Germany are offered a mammogram biennially as part of the German mammography screening program (MSP), which has been fully implemented since 2009. The participation rate per year among invited women is consistently reported to be around 50% [1]. This figure is often interpreted as indicating that only 50% of eligible women participate in the German MSP. However, such an interpretation assumes selective adherence, meaning that half of the invited women participate in each round while half never participate. A participation rate of 50% per year could, however, also arise under a different pattern, for example, if 25% of invited women participated in every round and 50% of eligible women participated only in every second round. In that case, the proportion of women participating at least once in the German MPS would be 75%. In contrast to selective adherence, this pattern is also referred to as sporadic adherence [2].

From a public health perspective, distinguishing between these adherence patterns is important because the implications for improving participation can differ substantially. If a 50% participation rate reflects selective adherence, the key challenge is how to reach the other half of the target population that never attends screening. If, by contrast, it reflects sporadic adherence the more relevant question is whether women need more information about the importance of regular participation.

A previous study of ours investigating the proportion of women participating at least once in mammography screening during the first 10 years of eligibility (i.e., age 50 to 59) indicated that participation in the German MSP is more consistent with sporadic than with selective adherence [3]. This suggests that the proportion of women who are (in principle) interested in engaging with the MSP is higher than the yearly participation rate implies. However, that study was limited to a single birth cohort and one data source, namely health claims data. Given the public health relevance of this topic, the present study extends our previous study by assessing the proportion of women who participated in the MSP at least once between age 50 and 60 (i) using different data sources, (ii) across different birth cohorts, and (iii) over a longer follow-up period (between age 50 and 63), and by describing (iv) the distribution of age at first MSP participation. Additionally (v), we determined the number of MSP participations.

## Materials and methods

### Data sources

We used two data sources: A) health claims data from four statutory health insurance providers (the so-called GePaRD data) covering ~ 20% population of the overall German population as well as 20% of the population of NRW, the largest federal state in Germany; and B) data from the Cancer Registry NRW. This data source includes information on MSP participation obtained from record linkage with screening units. The individual-level data were supplemented with aggregated information on deaths and population counts from the regional federal statistical office of NRW to identify a subgroup of MSP non-participants that could not be captured through the LKR NRW data alone. The resulting data cover the entire population of NRW (together forming the so-called LKR NRW data).

### Study population and study design

In the first part of the analyses, we included women born in 1959, i.e., women turning 50 in 2009, the first year with full roll-out of the MSP in Germany. We determined the cumulative probability of at least one MSP participation until 2019, i.e., the year these women turned 60 (max. follow-up: 11 years). We conducted these analyses based on GePaRD, GePaRD-NRW (for comparison with GePaRD as a whole) as well as the LKR NRW data.

In additional analyses using only GePaRD (for reasons of data availability), we compared the cumulative probability of at least one MSP participation between age 50 and 60 for different birth cohorts (women turning 50 in 2010, 2011 and 2012, i.e., birth cohorts born in 1960–1962; max. follow-up of 11 years). We also determined the cumulative probability of MSP participation between age 50 and 64 for the birth cohort of 1959 based on data until 2022 (max. follow-up: 14 years). For the birth cohorts 1959–1962, we determined the age distribution at first participation. Further, for women of these birth cohorts, we determined the number of participations over 11 years of follow-up.

In sensitivity analyses, we compared MSP participation between women turning 50 before 2018 and women turning 50 in 2018 or later. We restricted this analysis to the first five years of follow-up because data were available only until 2022. We conducted this sensitivity analysis because, since the end of 2017, revised information material provided with the invitation to screening has been used, offering women a decision aid now also addressing their preference for the frequency of screening [4, 5], which might have had an influence on participation.

### Data analyses

We determined the cumulative probability of at least one MSP participation (i.e., the proportion of women participating in the MSP at least once) for the respective birth cohorts using Kaplan Meier analyses. Since the health claims data only contain information on the birth year (rather than the month- and year-specific date of birth in the LKR NRW data), follow-up in this data source started January 1 of the year in which women turned 50 for each cohort. We followed up women until death, end of continuous insurance (GePaRD data), or December 31 of the respective study period, whichever came first. Regarding age at first MSP participation, we considered women participating at least once and determined the age distribution at their first participation according to the following age groups: 50–51, 52–53, 54–55, 56–57, and 58–60 years. To determine the frequency of MSP participation, we determined the respective proportions of women participating 0 to a maximum of 6 times, including only women with at least 11 years of follow-up (in a sensitivity analysis, all women were included irrespective of follow-up).

Analyses based on health claims data were conducted using SAS® Software (Version 9.4, SAS Institute Inc., Cary, NC, USA), and analyses based on LKR NRW data using R (Version 4.4.0 with packages: dplyr 1.1.4, writexl 1.5.0, survival 3.7.0, survminer 0.5.0, and patchwork 1.3.1).

## Results

In the 2009 cohort, 117,509 women were included in GePaRD, of whom 25,105 women were NRW residents, and 139,243 women were included in LKR NRW data. In GePaRD, 71.0% of women (GePaRD-NRW: 73.6%) participated at least once in the MSP until the end of 2019 (Table 1). In the LKR NRW data, the proportion with at least one participation between age 50 and 60 was 75.4%. As shown in Table 1 for GePaRD, the cumulative proportion with at least one participation until age 60 was stable at about 70% across different cohorts (2010: 70.4%, 2011: 70.1%, 2012: 69.7%). The cumulative probability of participating until age 53 was 57–58% (2009: 57.3%, 2010: 57.8%, 2011: 58.3%, 2012: 58.3%). When extending the follow-up in GePaRD for women turning 50 in 2009 until the end of 2022, the cumulative proportion with at least one participation was 72.6%.

**Table 1:** Cumulative probability of at least one MSP participation for different cohorts according to data source (GePaRD and LKR NRW)

	Cumulative probability of at least one participation
LKR NRW vs. GePaRD: Comparison of cohort 2009^a^ (follow-up until 2019)	
LKR NRW	75.4%
GePaRD	71.0%
GePaRD-NRW	73.6%
GePaRD: Extended follow-up for cohort 2009^a^	
Follow-up until 2020	71.5%
Follow-up until 2021	72.0%
Follow-up until 2022	72.6%
GePaRD: Additional cohorts^a^	
2010 (follow-up until 2020)	70.4%
2011 (follow-up until 2021)	70.1%
2012 (follow-up until 2022)	69.7%

*MSP* mammography screening program

^a^ Year in which women turned 50

**Fig. 1:**
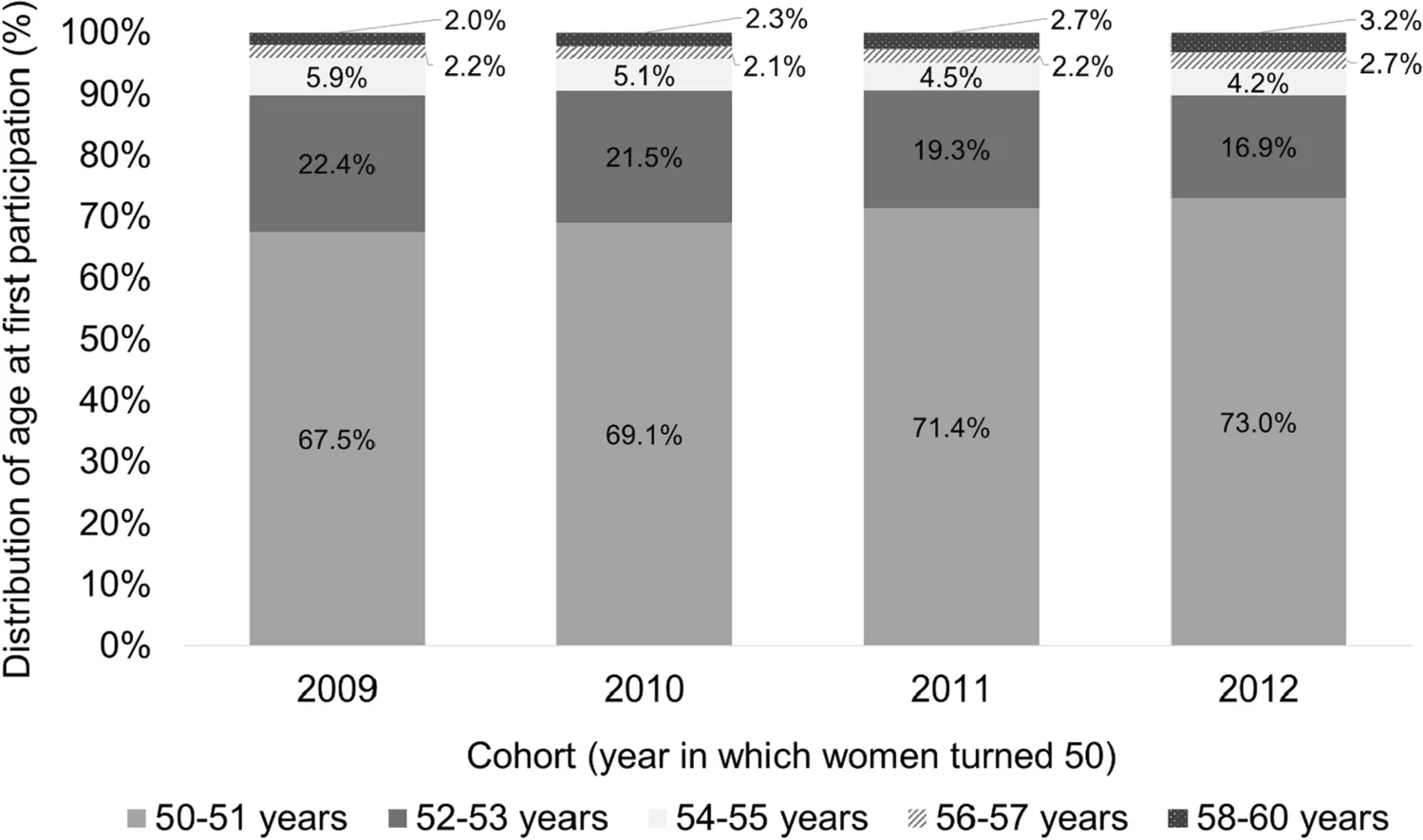
Distribution of age at first participation in women turning 50 in 2009–2012 with at least one MSP participation until the year they turned 60 (GePaRD)

Regarding age at first MSP participation among women participating at least once (n = 507,524 across birth cohorts 1959–1962), about 90% were between age 50 and 53 at first participation (Fig. 1).

Regarding the frequency of MSP participation among women participating at least once, 52.4% participated 1–4 times and 47.6% participated 5–6 times. When considering all women in the denominator and defining 5–6 participations during follow-up as regular participation, the proportions of non-participants, irregular participants and regular participants were 29.2, 37.1, and 33.7%, respectively (Supplementary Tables 1–2).

In the sensitivity analysis comparing participation in cohorts turning 50 before 2018 and those turning 50 from 2018 onwards, the cumulative probability of at least one MSP participation in the first five years of eligibility was 62.1% in the former cohort and 63.7% in the latter.

## Discussion

Using different data sources and considering different birth cohorts, our study consistently showed that about 70% of eligible women participated at least once in the German MSP between age 50 and 60. The vast majority of these women did so for the first time between age 50 and 53, which suggests that the decision to participate in the MSP is made in the first few years of eligibility. More than half of the women who participated at least once did not participate at the recommended regular intervals between age 50 and age 60.

The participation per year as reported by the coordinators of the program in Germany is only 50% [1]. If the proportion reported by the coordinators reflected a pattern of selective adherence, the results of our study on cumulative participation over 11 years would have shown a proportion of about 50%, which was not the case. Rather did we observe a cumulative participation of 70%. This, supported by our results on the number of participations, means that the participants in each year are composed of regular and irregular participants, i.e., participation in the German MSP is based on a sporadic adherence pattern.

This raises the question of whether the situation is similar in other countries that have an organized MSP implemented based on European Guidelines [6]. However, direct information on the proportion of regular and irregular participation from European countries is limited. In a study from the Capital Region of Denmark investigating longitudinal adherence between 2008 and 2017, 32.8% of women participated between 1 and 4 times in ten years [7]. A study from Norway investigating longitudinal adherence between 1996 and 2019 showed that 41.5% of women attended 1 to 9 of ten invitations and 26.6% attended 1 to 8 of ten invitations [8]. Accordingly, irregular participation does not seem to be an issue in Germany alone but given the scarcity of available data on longitudinal adherence patterns from other countries, it is difficult to draw concrete comparisons. Nevertheless, unlike Germany, many other countries reach the recommended participation of 70–75% per year [9].

To achieve this in Germany as well, our findings show that it would not necessarily be required to reduce the proportion of non-participants. It would also be sufficient if women who have participated only irregularly so far were to become regular participants.

While interest in the program among eligible women in Germany seems to be relatively high (70% of them engage with the program at least once), not all women who initially engage with the program keep participating. Investigation of reasons for irregular participation should therefore have a high priority In the German MSP. This could be achieved, for example, through qualitative and quantitative studies—such as focus groups and representative population surveys—that examine the reasons for irregular participation and could lay the groundwork for intervention studies. There was a survey on reasons of non-participation conducted in 2018 [10], which could be updated and extended in scope by also considering women participating irregularly in the MSP.

Our findings regarding age at first participation highlight the importance of providing appropriate information at the start of eligibility. Generally, the information material provided with the invitation to the German MSP is very comprehensive but if it turns out that the reasons for the two-year interval are not properly understood, adjustments could be considered, possibly also with regard to providing the information in plain language. On the other hand, in a survey conducted between 2012 and 2013 among 336 women who were close to turning 50, Gummersbach et al. found that the information material played only a minor role in the women’s readiness to participate; only 3.6% of the women considered the written material as the most important factor in their decision, while for 48.2% the recommendation from a physician was the most important factor [11]. Although this study was conducted more than 10 years ago, it may still be justified to further consider the role of physicians, particularly gynecologists, in the decision-making process and to plan implementation studies examining how they could be integrated into this process. Since the end of 2017, revised information material has been used offering women a decision aid which also addresses their preference regarding the frequency of screening [4, 5]. In our sensitivity analysis comparing participation in women turning 50 before 2018 and from 2018 onwards, we did not find evidence of changes in participation after the new information material became available. However, since we did not investigate the frequency of screening in this comparison, the possible effects on the regularity of participation should be monitored once sufficient follow-up after 2018 is available.

Our study has strengths and limitations. Methodologically, it illustrates that the longitudinal perspective could be a useful addition to the most frequently assessed participation per year, i.e., the cross-sectional perspective, since the longitudinal perspective makes it possible to determine the proportion of women who do not participate at all, participate irregularly, or participate regularly. Such analyses may thus have the potential to complement existing screening measures, but should certainly not replace them. Our approach of using different data sources and comparing different birth cohorts enabled us to assess the cumulative participation in the German MSP with high robustness. Unlike in the LKR NRW data, only the year in which women turned 50 was available in health claims data, but this difference had no relevance regarding our main findings on the probability of participating at least once by the end of follow-up. This limitation also affected the categorization of age at first participation, but similarly had no impact on our conclusion that the vast majority of participating women had a mammography already within a few years after eligibility, i.e., after turning 50.

In conclusion, our study shows that the proportion of women who are (in principle) interested in the German MSP is higher than the yearly participation rate implies. The longitudinal perspective thus provided important additional information for health decision-makers because it revealed the high proportion of irregular participants. Studies are needed to investigate reasons for irregular participation and how they could be considered in future implementation strategies.

## Supplementary Information

Below is the link to the electronic supplementary material.

Supplementary file1 (DOCX 16 KB)

## Data Availability

As we are not the owners of the data, we are not legally entitled to grant access to the data of the so-called German Pharmacoepidemiological Research Database. In accordance with German data protection regulations, access to the data is granted only to employees of the Leibniz Institute for Prevention Research and Epidemiology – BIPS on the BIPS premises and in the context of approved research projects. Third parties may only access the data in cooperation with BIPS and after signing an agreement for guest researchers at BIPS. The data from the cancer registry were also requested for health reporting and research projects in accordance with Sects. 23 and 24 of the North Rhine-Westphalia Cancer Registry Act, and were approved by the responsible Institutional Review Board of the Cancer Registry of North Rhine-Westphalia. Therefore, these data also cannot be made available. Additional aggregated data of the State Agency Information and Technology North Rhine-Westphalia (IT.NRW) were also provided upon request.

## Abbreviations

GePaRD: German Pharmacoepidemiological Research Database
MSP: Mammography screening program
NRW: North-Rhine Westphalia
LKR NRW: Cancer Registry North-Rhine Westphalia
