# Supplementary material for: Participation in the German mammography screening program: about 70% of women participate at least once between age 50 and 60
Source: Cancer Causes Control. 2026 Nov 12;37(12):195. doi: 10.1007/s10552-026-02278-2 (PMC13759708; doi:10.1007/s10552-026-02278-2)
Supplement: Supplementary file 1 [file 10552_2026_2278_MOESM1_ESM.docx]

**Supplementary Information**

**Participation in the German mammography screening program: About 70% of women participate at least once between age 50 and 60**

Miriam Heinig^1^, Laura Buschmann^2^, Florian Oesterling^3^, Bianca Kollhorst^4^, Jonas Czwikla^5^, Heike Minnerup^2^, André Karch^2^, Ulrike Haug^1,6^

1 Department of Clinical Epidemiology, Leibniz Institute for Prevention Research and Epidemiology – BIPS, Bremen, Germany

2 Institute of Epidemiology and Social Medicine, University of Münster, Münster, Germany

3 Cancer Registry North Rhine-Westphalia, Bochum, Germany

4 Department of Statistical Methods in Epidemiology, Leibniz Institute for Prevention Research and Epidemiology – BIPS, Bremen, Germany

5 Julius-Maximilians-Universität Würzburg, Institute for Clinical Epidemiology and Biometry, Würzburg, Germany

6 Faculty of Human and Health Sciences, University of Bremen, Bremen, Germany

**Correspondence:**

Dr. Miriam Heinig
Leibniz Institute for Prevention Research and Epidemiology – BIPS
Department of Clinical Epidemiology
Achterstraße 30, 28359 Bremen, Germany
Phone: +49-(0) 421-218-56881; Fax: +49-(0)421-218-56821
E-Mail: heinig@leibniz-bips.de

**Supplementary Table 1** Number of MSP participations within 11 years of follow-up (study population: women turning 50 between 2009 and 2012 with at least 11 years of follow-up (GePaRD data).

| **Total number of participations** | **N=408,549** |
| --- | --- |
|  | **Proportion (%)** |
| 0 | 29.2% |
| 1 | 8.3% |
| 2 | 7.0% |
| 3 | 8.2% |
| 4 | 13.6% |
| 5 | 27.7% |
| 6 | 6.0% |
| **Pattern of participation^a^** (considering all women in the denominator) | |
| 0 times | 29.2% |
| 1-4 times | 37.1% |
| 5-6 times* | 33.7% |
| **Pattern of participation^a^** (considering only women with at least one participation in the denominator) | |
| 1-4 times | 52.4% |
| 5-6 times | 47.6% |

^a^ Since the invitation date and date of birth are not in the data, the proportion of women who participated regularly in screening could not be determined precisely; however, it can be assumed that women who participated 5–6 times during the follow-up period participated in each offered round (regular participants).

**Supplementary Table 2** Number of MSP participations within 11 years of follow-up among women who participated at least once (study population: women turning 50 between 2009 and 2012 at least one MSP participation, inclusion irrespective of follow-up time (GePaRD data).

| **Total number of participations** | **N=507,524** |
| --- | --- |
|  | **Proportion (%)** |
| 0 | 34.1% |
| 1 | 10.2% |
| 2 | 7.9% |
| 3 | 8.4% |
| 4 | 11.9% |
| 5 | 22.6% |
| 6 | 4.9% |
| **Pattern of participation^a^** (considering all women in the denominator) | |
| 0 times | 29.2% |
| 1-4 times | 38.4% |
| 5-6 times* | 27.4% |
| **Pattern of participation^a^** (considering only women with at least one participation in the denominator) | |
| 1-4 times | 58.3% |
| 5-6 times | 41.7% |

^a^ Since the invitation date and date of birth are not in the data, the proportion of women who participated regularly in screening could not be determined precisely; however, it can be assumed that women who participated 5–6 times during the follow-up period participated in each offered round (regular participants).
